# Reduced microRNA-503 expression augments lung fibroblast VEGF production in chronic obstructive pulmonary disease

**DOI:** 10.1371/journal.pone.0184039

**Published:** 2017-09-07

**Authors:** Jun Ikari, Amy J. Nelson, Jannah Obaid, Alvaro Giron-Martinez, Kumiko Ikari, Fumihiko Makino, Shunichiro Iwasawa, Yoko Gunji, Maha Farid, Xingqi Wang, Hesham Basma, Dawn Demeo, Carol Feghali-Bostwick, Olaf Holz, Klaus Rabe, Xiangde Liu, Stephen I. Rennard

**Affiliations:** 1 Pulmonary, Critical Care, Sleep and Allergy Medicine, University of Nebraska Medical Center, Omaha, Nebraska, United States of America; 2 Respirology, Graduate School of Medicine, Chiba University, Chiba, Japan; 3 Channing Laboratory, Harvard Medical School, Boston, Massachusetts, United States of America; 4 Division of Rheumatology & Immunology, Department of Medicine, Medical University of South Carolina, Charleston, South Carolina, United States of America; 5 Fraunhofer ITEM, Member of the German Center for Lung Research (DZL, BREATH), Hannover, Germany; 6 Airway Research Center North (ARCN), Lungen Clinic Grosshansdorf, Member of the German Center for Lung Research, Grosshansdorf, Germany; Institut de Pharmacologie Moleculaire et Cellulaire, FRANCE

## Abstract

Alterations in microRNA (miRNA) expression may contribute to COPD pathogenesis. In COPD, lung fibroblast repair functions are altered in multiple ways, including extracellular mediator release. Our prior study revealed miR-503 expression is decreased in COPD lung fibroblasts, although the exact role played by miR-503 is undetermined. The current study examined a role of miR-503 in cytokine, growth factor and fibronectin production by lung fibroblasts from patients with and without COPD. Primary adult lung fibroblasts were isolated from patients with or without COPD. MiR-503 expression and interleukin (IL)-6, -8, PGE_2_, HGF, KGF, VEGF and fibronectin release were examined with or without inflammatory cytokines, IL-1β and tumor necrosis factor (TNF)-α. MiR-503 expression was decreased in COPD lung fibroblasts. The expression of miR-503 was positively correlated with %FVC, %FEV1, and %DLco as well as IL-6, -8, PGE_2_, HGF, KGF, and VEGF in the absence or presence of IL-1ß/TNF-α. In addition, IL-8 and VEGF release from COPD lung fibroblasts were increased compared to those from control. Exogenous miR-503 inhibited VEGF release from primary adult and fetal lung fibroblasts but not IL-8 release. As expected, COPD fibroblasts proliferated more slowly than control fibroblasts. MiR-503 did not affect proliferation of either control or COPD lung fibroblasts. MiR-503 inhibition of VEGF protein production and mRNA was mediated by direct binding to the 3’ untranslated region of VEGF mRNA. Endogenous miR-503 was differently regulated by exogenous stimulants associated with COPD pathogenesis, including IL-1ß/TNF-α, TGF-ß1 and PGE_2_. Endogenous miR-503 inhibition augmented VEGF release by IL-1ß/TNF-α and TGF-ß1 but not by PGE_2_, demonstrating selectivity of miR-503 regulation of VEGF. In conclusions, reduced miR-503 augments VEGF release from lung fibroblasts from patients with COPD. Since VEGF contributes to disturbed vasculature in COPD, altered miR-503 production might play a role in modulating fibroblast-mediated vascular homeostasis in COPD.

## Introduction

Chronic obstructive pulmonary disease (COPD) was projected to be the third leading cause of death worldwide by 2020 [1], but achieved this distinction several years early [WHO Factsheet. The top 10 causes of death. 2014.]. COPD is characterized by small airway obstruction and emphysema, leading to airflow limitation. A principal cause of COPD is smoking that induces chronic inflammation, tissue destruction and inhibits tissue repair in the lungs [2]. Levels of pro-inflammatory cytokines, such as interleukin (IL)-1β and tumor necrosis factor (TNF)-α are increased in COPD and are targets for anti-inflammatory treatments [3]. Lung fibroblasts compose 20–40% of alveolar cells and contribute to the repair, maintenance and integration of lung structures [4–7]. Recent studies revealed that, in COPD, functions of lung fibroblasts are altered in multiple ways [8–11] and altered lung fibroblast production of inflammatory cytokines, growth factors and fibronectin could contribute the pathogenesis of COPD [6].

MicroRNAs (miRNAs) are a class of small non-coding RNAs that regulate the translation of target genes. MiRNA is involved in diverse biological processes including organ development, growth control, differentiation and cell death [12, 13]. Although miRNA expression is varied in pulmonary diseases, including COPD, the precise role of miRNAs, especially in specific cell types, is not fully understood [14, 15]. Previously, by using miRNA-microarray analysis, we identified that expression of several miRNAs was consistently altered in COPD lung fibroblasts cultured *in vitro* and that this was independent of culture density or cell passage (between passages three and eight). Of these, miR-503 expression was decreased in COPD lung fibroblasts with or without IL-1β and TNF-α stimulation [16]. Although, miR-503 is abundantly expressed in the lung [17], the role of miR-503 and its target genes in lung fibroblasts has not been examined.

Thus, the current study sought to examine a functional role for miR-503 in lung fibroblasts from patients with COPD and control. We elucidated that miR-503 is consistently decreased in COPD lung fibroblasts and that the level of miR-503 expression is associated with COPD severity. We further examined associations between miR-503 expression and fibroblast production of IL-6, -8, prostaglandin (PG)E2, hepatocyte growth factor (HGF), keratinocyte growth factor (KGF), vascular endothelial growth factor (VEGF) and fibronectin, all of which have been suggested to contribute to COPD pathophysiology [8, 11, 18–21]. Among these, decreased miR-503 was associated with augmented VEGF production by COPD lung fibroblasts with and without stimulation of exogenous IL-1β and TNF-α. Moreover, MiR-503 repressed VEGF by direct binding to the 3’-untranslated region (3’-UTR) of VEGF mRNA in human lung fibroblasts. Finally, we examined the physiological regulation of miR-503 expression in lung fibroblasts and its role in VEGF release in response to mediators. VEGF is a potent growth factor essential for maintaining pulmonary vasculature, which is disturbed in COPD [22]. Thus, the results of the current study suggests that miR-503 plays a role in modulating fibroblast-mediated vascular homeostasis in COPD.

## Materials and methods

### Materials

Dulbecco’s Modified Eagle Medium (DMEM), fetal calf serum (FCS), Opti-MEM, Lipofectamine 2000 and Trizol were from Invitrogen (Grand Island, NY, USA); 3,3,5,5-tetramethylbenzidine (TMB) and prostaglandin E_2_ (PGE_2_) (10^−7^ M) from Sigma-Aldrich (St. Louis, MO, USA); hsa-miRNA-503 Mimic, hsa-miRNA-503 Inhibitor and microRNA Negative Control (25 nM) were from Dharmacon (Lafayette, CO, USA); anti-human VEGF antibody (AF293-NA), biotinylated anti-human VEGF antibody (BAF293), anti-human IL-8 antibody (MAB208), biotinylated anti-human IL-8 antibody (BAF208), recombinant human TGF-ß1, IL-1ß, TNF-α, Human IL-6 DuoSet ELISA kit, Human HGF DuoSet ELISA kit, Human and KGF/FGF-7 DuoSet ELISA were from R&D Systems (Minneapolis, MN, USA); horseradish peroxidase (HRP)-streptavidin conjugate was from Zymed Laboratories (South San Francisco, CA, USA); and Prostaglandin E_2_ (PGE_2_) assay kit (EIA) was from Cayman (Ann Arbor, MI, USA).

### Cell culture

Human fetal lung fibroblasts (HFL-1; lung, diploid, human) were purchased from the American Type Culture Collection (CCL-153; Rockville, MD, USA). Primary lung fibroblasts from 19 subjects without COPD (controls) and 18 subjects with COPD were included (Table 1). Lung specimens from 5 subjects with COPD and 6 subjects without COPD were obtained from lungs resected for lung tumor. Lung specimens from 13 subjects with COPD were obtained from lungs removed for transplantation, and from 13 subjects without COPD from unused donor lungs harvested for potential transplantation at the University of Pittsburgh. Acquisition of samples was approved by the Human Studies Committee of the Medical Board of the State of Schleswig-Holstein, or that of the University of Pittsburgh. All subjects provided written informed consent for research. Primary fibroblast cultures were initiated from normal-appearing areas of the pulmonary parenchyma that were free of the pleura or large airways and were cultured as described elsewhere [8, 23]. Then, 2.5 x 10^5^ cells were plated in 60-mm culture dishes with fetal calf serum (FCS) containing DMEM. Medium was changed after one day and then every two days until five days. For stimulation, lung fibroblasts were cultured in DMEM with 10% fetal calf serum (FCS) for 2 days. Culture media were then changed to DMEM without serum for 2 hours, following which serum-free DMEM supplemented with or without IL-1β and TNF-α, TGF-ß1 or PGE_2_ was added. The cells were then cultured for an additional 48 hours.

**Table 1:** Clinical features of subjects.

	Control	COPD	*P* value
Number of subjects	19	18	
Age (Year)	53.8 ± 4.9	61.3 ± 1.6	NS
Gender (M/F)	10/9	11/7	NS
Smoking history (Non-smoker/Smoker/NA)	7/8/4	0/18/0	< 0.001
Smoking (pack-years)	24.2 ± 13.1	45.5 ± 4.9	< 0.05
GOLD stage (I/II/III/IV)	0/0/0/0	0/2/10/6	
FVC * (% of predicted value)	104.2 ± 7.1	76.0 ± 5.5	< 0.01
FEV1 ^†^ (% of predicted value)	101.0 ± 8.1	33.5 ± 3.9	< 0.0001
DLco ^‡^ (% of predicted value)	87.5 ± 9.8	38.2 ± 3.8	< 0.0001
mPAP ^§^ (mmHg)	NA	25.8 ± 3.2	
6MWD ^a^ (m)	NA	270.0 ± 25.3	

Definition of Abbreviations: COPD; chronic obstructive pulmonary disease, NS; not significant, NA; data not available, GOLD; Global Initiative for Chronic Obstructive Lung Disease, DLco; diffusion capacity for carbon monoxide, mPAP; mean pulmonary arterial pressure, 6MWD; 6-minute walk distance. Values are mean ± SEM, except for a number of subjects, gender, smoking history and GOLD stage (number).

* FVC % predicted values are from 6 subjects of control and 18 subjects of COPD.

^†^ FEV1% predicted values are from 6 subjects of control and 18 subjects of COPD.

^‡^ DLco % predicted values are from 5 subjects of control and 14 subjects of COPD.

^§^ mPAP values are from 12 subjects of COPD.

^a^ 6MWD values are from 12 subjects of COPD.

### Transfection of microRNA mimic or inhibitor

Total 1.5 × 10^5^ cells were plated in each well of 6-well plates without antibiotics and amphotericin B and cultured for 24 hours. Cells were transfected with negative control miRNA, miR-503 Mimic or miR-503 Inhibitor (25 nM, respectively). After 6 hours transfection, cells were further cultured for 24 hours and then stimulated by various reagents for an additional 48 hours.

### ELISA and EIA

VEGF concentrations in the supernatants of cultures were measured by ELISA. Briefly, ELISA plates were coated with monoclonal anti-human VEGF antibody at 4°C overnight. Plates were washed three times, and then standards or samples were applied to individual wells and incubated at room temperature for 2 hours, followed by washing and the application of biotinylated anti-human VEGF antibody for 1 hour at room temperature. After washing, HRP—streptavidin conjugate was added for 1 hour at room temperature. After a final wash, bound HRP was detected with TMB, as described elsewhere [24]. The reaction was stopped with 1 M H2SO4, and quantified at 450 nm with a microplate reader (Bio-Rad, Hercules, CA, USA). IL-6, IL-8, PGE_2_, HGF, KGF production from cells was determined by ELISA or EIA following the manufacturer’s instructions. Fibronectin release was determined by ELISA as described elsewhere [8].

### Real time PCR

Total RNA was isolated by Trizol reagent and transcribed to complementary DNA by using High-Capacity cDNA Reverse Transcription Kits (Applied Biosystems, Foster City, CA, USA). Both miR-503 specific primers from the TaqMan MicroRNA Assays and TaqMan MicroRNA Reverse Transcription Kit were used for reverse transcription of miR-503 (Applied Biosystems, Foster City, CA, USA). Real time PCR for VEGF mRNA and miR-503 were conducted with TaqMan Gene Expression Assays and the ABI Prism 7500 (Applied Biosystems) following the manufacturer’s instructions. As an internal control, the ribosomal RNA (18s rRNA) control kit (Applied Biosystems) was used.

### Luciferase assay

HFL-1 cells cultured in monolayer were co-transfected with a VEGF 3’ UTR-LUC construct, control vector (HmiT018477-MT01, CmiT000001-MT01; Rockville, MD, USA), miR-503 mimic, and control miRNA. Cell layers were harvested 48hr after transfection, and luciferase activity was analyzed by Dual-Luciferase Reporter Assay System (Promega) and luminometer (MicroLumat Plus-LB96V, EG&G Berthold, Bad Wildbad, Germany) as described elsewhere [10].

### Statistical analysis

Data are expressed as means ± SE. Chi-square test or Fisher's exact test was used to evaluate statistical differences between the proportions in the two groups. Comparisons between the two groups of subjects were analyzed by using the two-tailed Mann-Whitney test. For evaluation of experiments within a group where paired samples were available, the Wilcoxon test was used. Spearman’s rank analysis was conducted to calculate correlation coefficients between two parameters. All the available subjects were included in the analysis. However, clinical data from control subjects was not fully available, because a subset of control specimens was from unused potential lung transplant donors and not all parameters were available. Furthermore, since cells from older subjects and from subjects with COPD grow slowly, sufficient materials were not obtained for all experiments. However, no available data were omitted from the statistical analyses. Probability values of < 0.05 were considered significant.

## Results

### Clinical features of study subjects

The clinical features of subjects without COPD (control) and with COPD are shown in Table 1. The number of smokers, and smoking history differed between control and COPD subjects. The patients with COPD were characterized with moderate to severe COPD, according to the criteria of the Global Initiative for Chronic Obstructive Lung Disease (GOLD). Subjects with COPD manifested the expected clinical features, including reduced FVC, FEV1, and diffusion capacity for carbon monoxide (DLco). Average mean pulmonary arterial pressure (mPAP) of subjects with COPD was more than 25 mmHg, which is consistent with pulmonary hypertension. As 13 of the control subject lung cells were obtained from donor lungs that were unusable for lung transplantation, lung function data were not available. However, there was no history of COPD in these subjects.

### MiR-503 expression is decreased in COPD lung fibroblasts with and without IL-1ß and TNF-α

**Fig 1:**
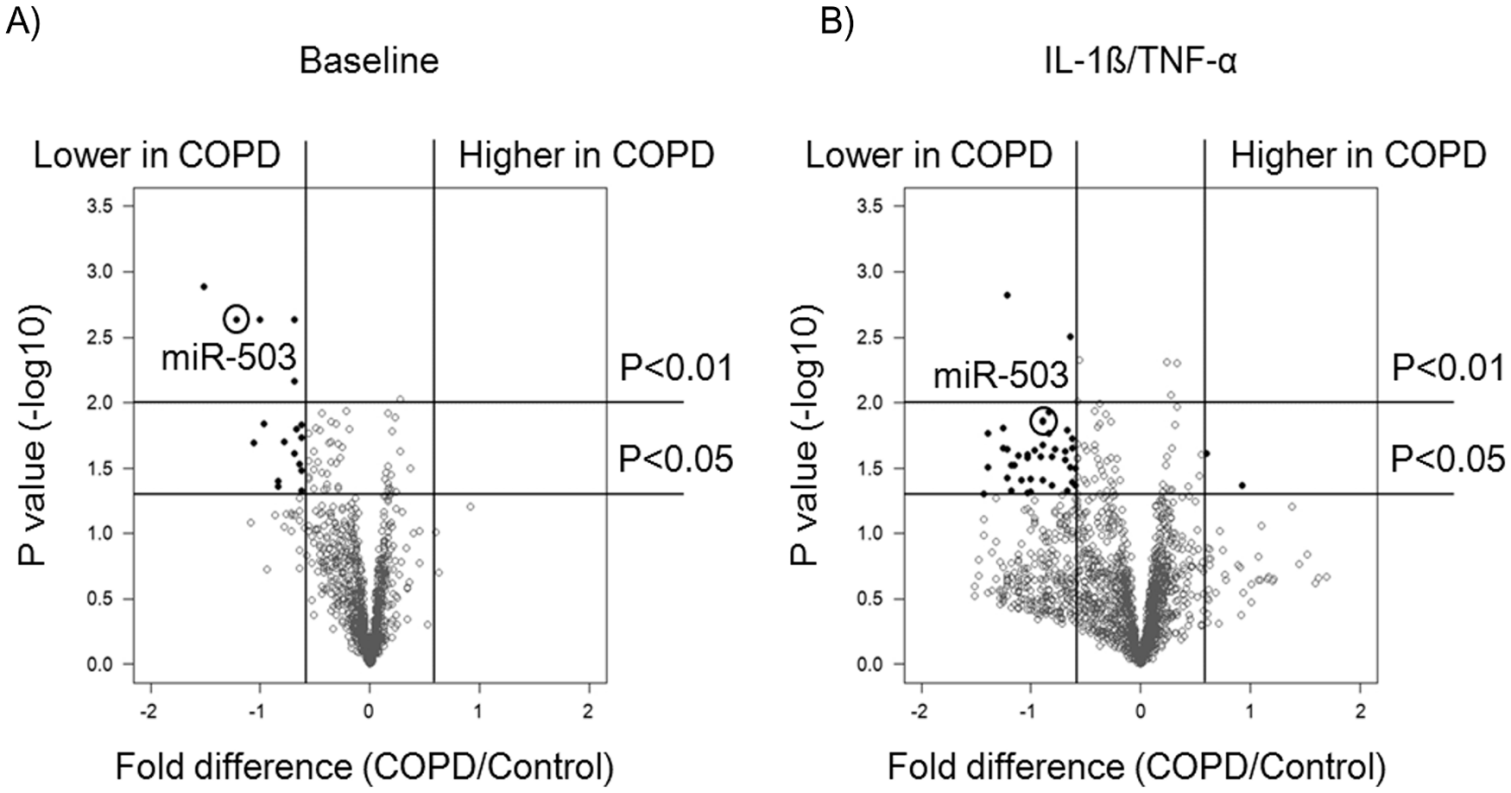
Differential miRNA expression between chronic obstructive pulmonary disease (COPD) and control lung fibroblasts in the absence or presence of IL-1ß and TNF-α. Control (n = 5) and COPD (n = 5) lung fibroblasts were cultured with 10% FCS containing DMEM for 2 days, after which cells were cultured with either (A) serum free DMEM (baseline) or (B) IL-1ß and TNF-α (1ng/ml) for 24 hours. Total RNA was extracted and miRNA microarrays were performed. Volcano plot of array data shows fold changes in miRNA expression between control and COPD lung fibroblasts, in which more than 1.25 fold change and with p value less than 0.05 were shown in black dots. MiR-503 is circled. Vertical axis: *p* value (-log10), Horizontal axis: fold difference of miRNA expression (COPD/control) expressed as log 2 value.

**Fig 2:**
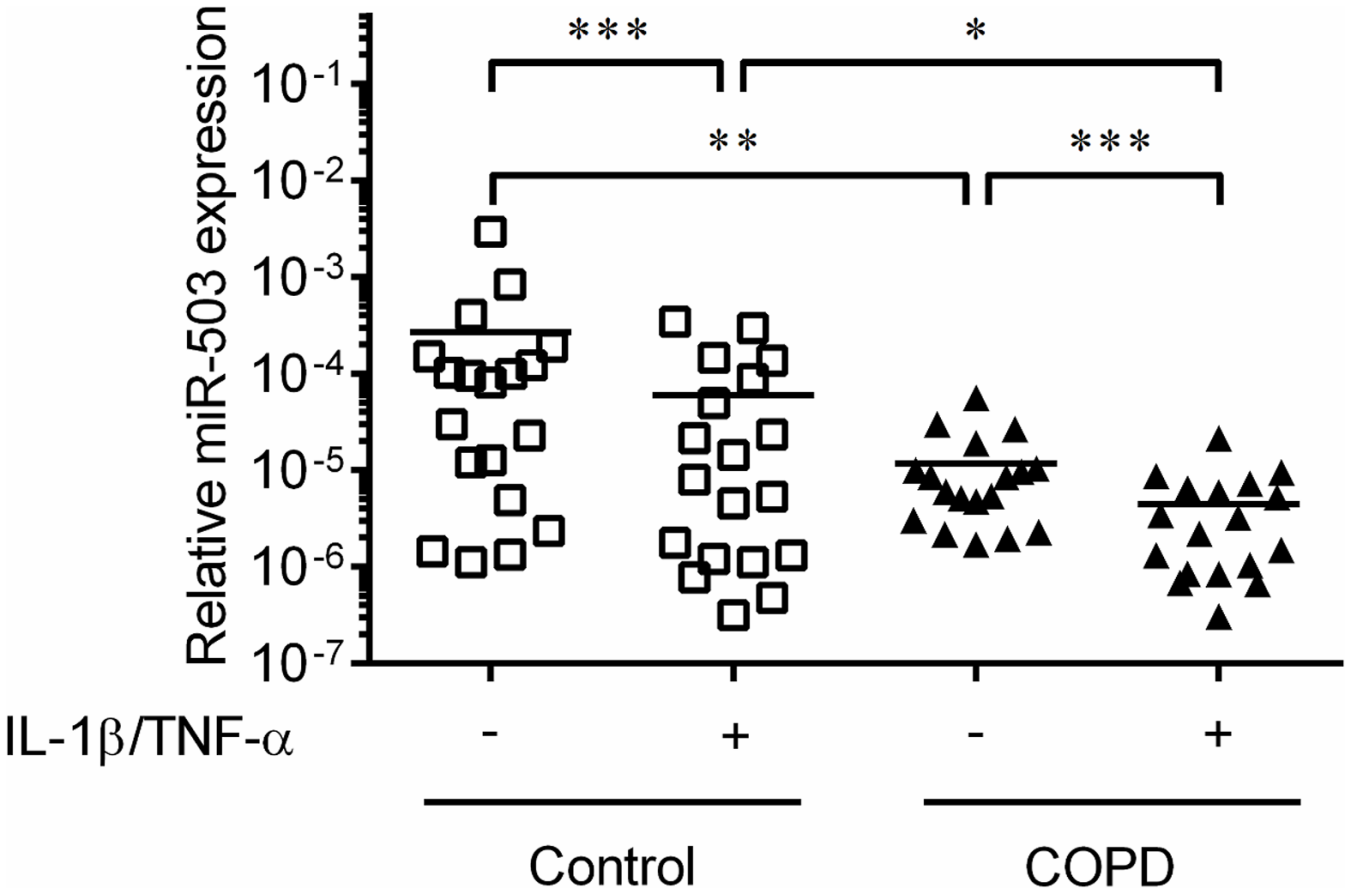
MiR-503 expression in COPD and control lung fibroblasts in the absence or presence of IL-1ß and TNF-α. Control (White square, n = 19) and COPD (Black triangle, n = 18) lung fibroblasts were cultured with 10% FCS containing DMEM for 2 days, after which the medium was changed to DMEM in the absence and presence of IL-1ß and TNF-α (1 ng/ml). After 1 day, total RNA was extracted from the cultured cells. MiR-503 expression was examined by real time qPCR. Vertical axis: level of miR-503 expression, expressed as fold of 18s-rRNA values in the same sample. Horizontal axis: culture condition. *p < 0.05, **p < 0.01, ***p < 0.001.

In a previous study, we explored the effect of culture conditions on miRNA expression in primary adult lung fibroblasts from patients with and without COPD that included five subjects in each group [16]. In order to identify potentially differentially expressed miRNAs, we performed a volcano plot analysis of those data and identified miR-503 as an miRNA that consistently differed between COPD and control lung fibroblasts either in the absence or presence of IL-1ß and TNF-α (Fig 1A and 1B). Accordingly, we replicated the miR-503 expression in primary adult lung fibroblasts by quantitative real-time PCR (qPCR) with and without IL-1ß and TNF-α in 18 subjects with and 19 subjects without COPD. These studies revealed that miR-503 expression was significantly decreased in COPD lung fibroblasts as compared to that of control lung fibroblasts both in the presence and absence of IL-1ß and TNF-α (*p* < 0.01 and < 0.05, respectively). Furthermore, IL-1ß and TNF-α significantly suppressed miR-503 expression in both control and COPD lung fibroblasts (*p* < 0.001) (Fig 2).

### The expression of miR-503 in lung fibroblasts correlates with lung function

**Fig 3:**
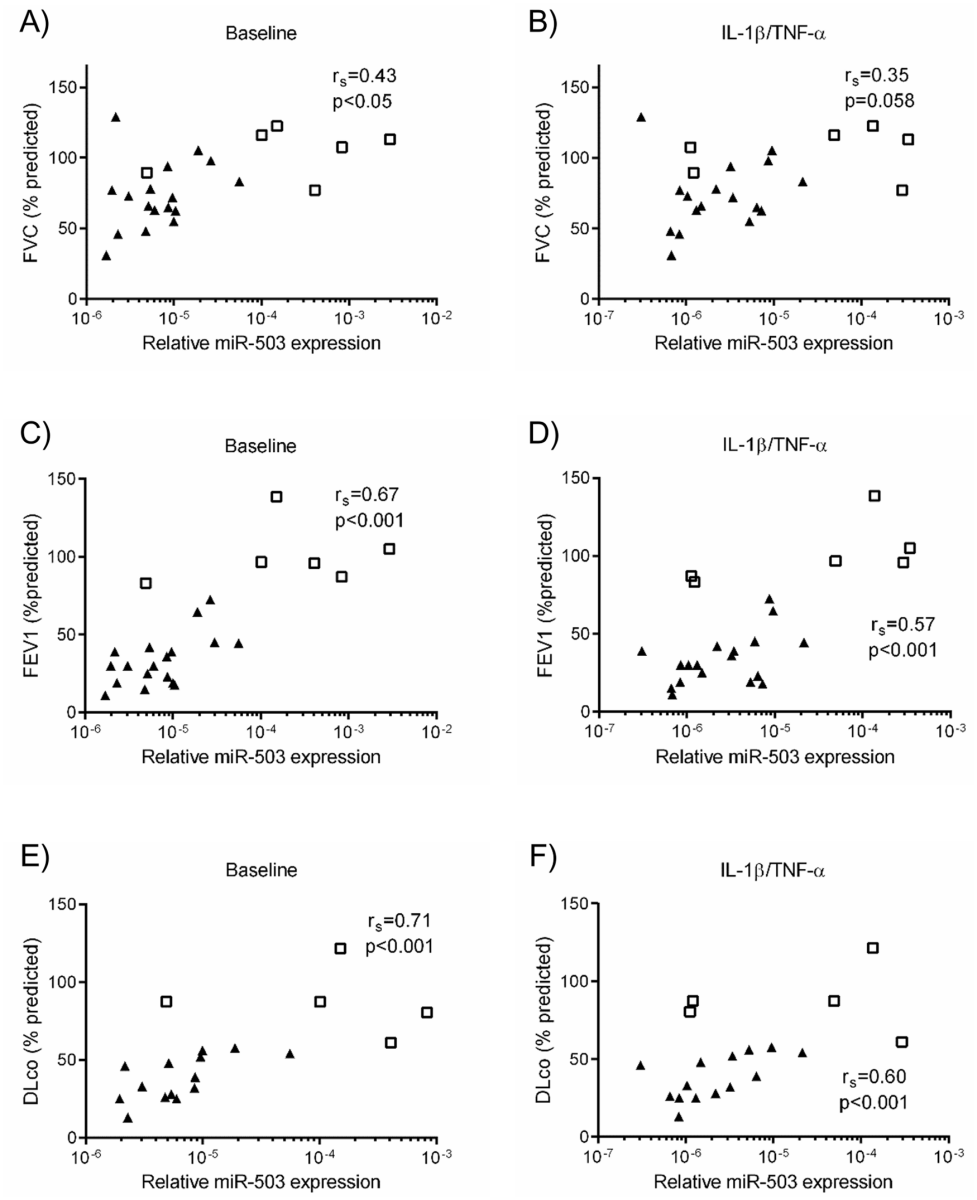
Correlations between lung function and miR-503 expression in control and COPD lung fibroblasts cultured with or without IL-1ß and TNF-α. Control (n = 19) and COPD (n = 18) lung fibroblasts were cultured with 10% FCS containing DMEM for 2 days, after which the medium was changed to DMEM in the absence (baseline) or presence of IL-1ß/TNF-α (1 ng/ml). After 1 day, total RNA was extracted from the cultured cells. Correlation between miR-503 expression in lung fibroblasts and %FVC (Control (n = 6) and COPD (n = 17)) ((A) baseline, (B) IL-1ß/TNF-α), %FEV1 (Control (n = 6) and COPD (n = 18)) ((C) baseline, (D) IL-1ß/TNF-α), % DLco (Control (n = 5) and COPD (n = 14)) ((E) baseline, (F) IL-1ß/TNF-α) were shown. White square: control, Black triangle: COPD. Horizontal axis: level of miR-503 expression, expressed as fold of 18s-rRNA values. The correlation was calculated by Spearman’s correlation test.

Since altered functions of lung fibroblasts are associated with clinical manifestations in patients with COPD [8, 10], we examined the correlation between miR-503 expression in adult lung fibroblasts and lung function and pulmonary artery pressure: FVC, FEV1 and DLco (all as %predicted) and mPAP. MiR-503 expression in lung fibroblasts was positively correlated with %FVC, %FEV1, %DLco and negatively correlated with mPAP when assessed in the absence or presence of IL-1ß and TNF-α (Fig 3A–3F, S1A and S1B Fig). In addition, when only the subjects from COPD were analyzed, miR-503 was positively correlated with %FEV1 (control: *p* < 0.05, rs = 0.54, IL-1ß/TNF-α: *p* < 0.05, rs = 0.54) and %DLco (control: *p* < 0.01, rs = 0.70, IL-1ß/TNF-α: *p* < 0.01, rs = 0.68) but not with %FVC (control: *p* = 0.22, rs = 0.31, IL-1ß/TNF-α: *p* = 0.19, rs = 0.33) (data not shown). MiR-503 expression was not related to age, amount of smoking or 6-minute walk distance (6MWD) (S1C–S1H Fig). Thus, miR-503 expression in lung fibroblasts significantly correlated with lung function measures that assess the severity of COPD.

### Cytokines, growth factors and ECM production and their correlations with miR-503 expression in primary adult lung fibroblasts

**Fig 4:**
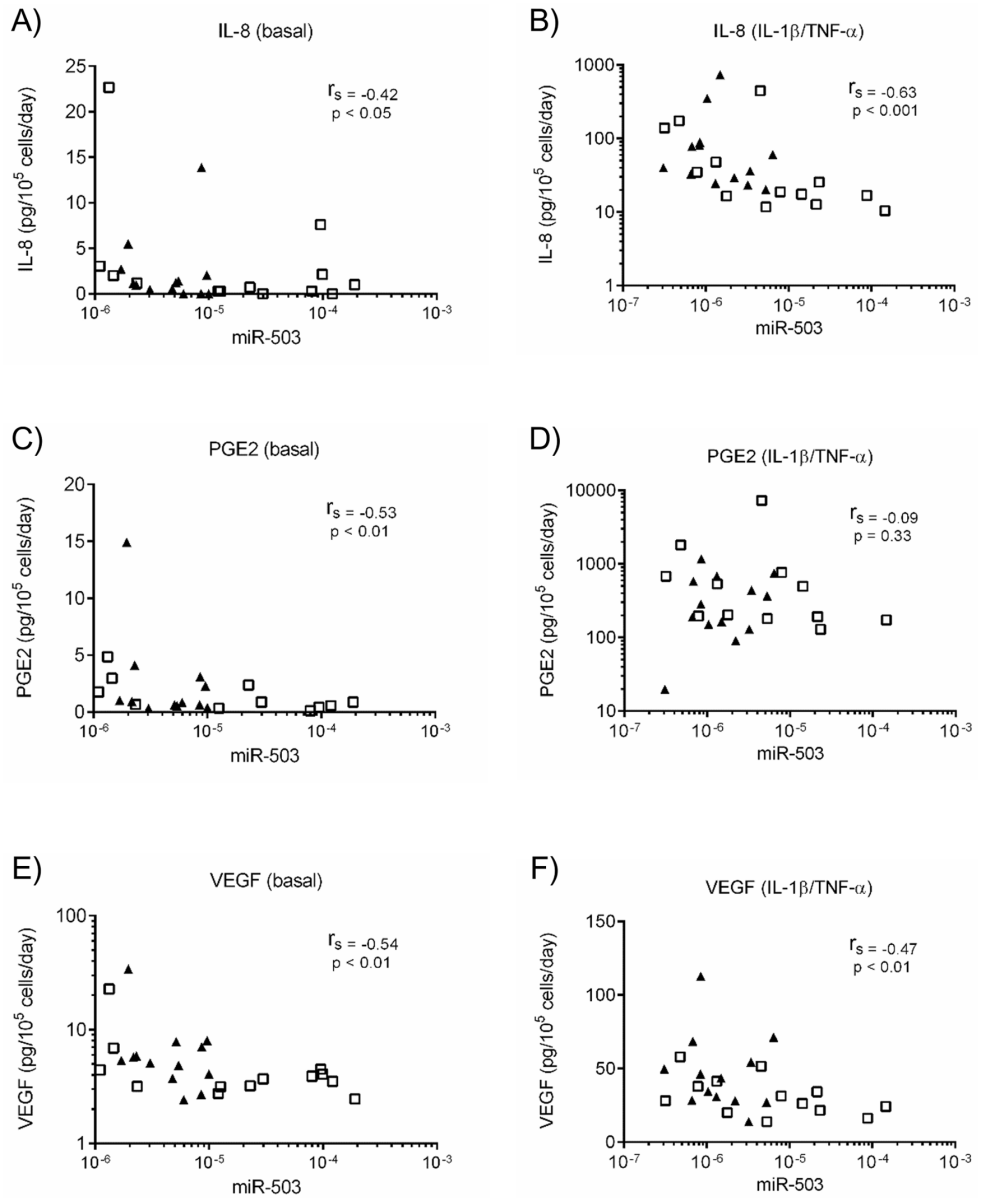
IL-8, PGE2, and VEGF release and their correlations with miR-503 expression in COPD and control lung fibroblasts. Control (n = 13) and COPD (n = 13) lung fibroblasts were cultured with 10% FCS containing DMEM for 2 days, after which the medium was changed to DMEM in the absence and presence of IL-1ß and TNF-α (1 ng/ml). After 1 day, the cell layer was harvested and miR-503 expression was examined by real-time qPCR. IL-8, PGE2, and VEGF release in the cultured medium were examined by ELISA or EIA. The correlation between miR-503 expression and IL-8 ((A) baseline, (B) IL-1ß/TNF-α), PGE2 ((C) baseline, (D) IL-1ß/TNF-α), and VEGF ((E) baseline, (F) IL-1ß/TNF-α) release of the same sample are shown. White square: control, Black triangle: COPD. Vertical axis: IL-8, PGE2, and VEGF release, respectively (pg per 10^5^ cells per 1 day). Horizontal axis: level of miR-503 expression, expressed as fold of 18s-rRNA values. The correlation was calculated by Spearman’s correlation test.

**Fig 5:**
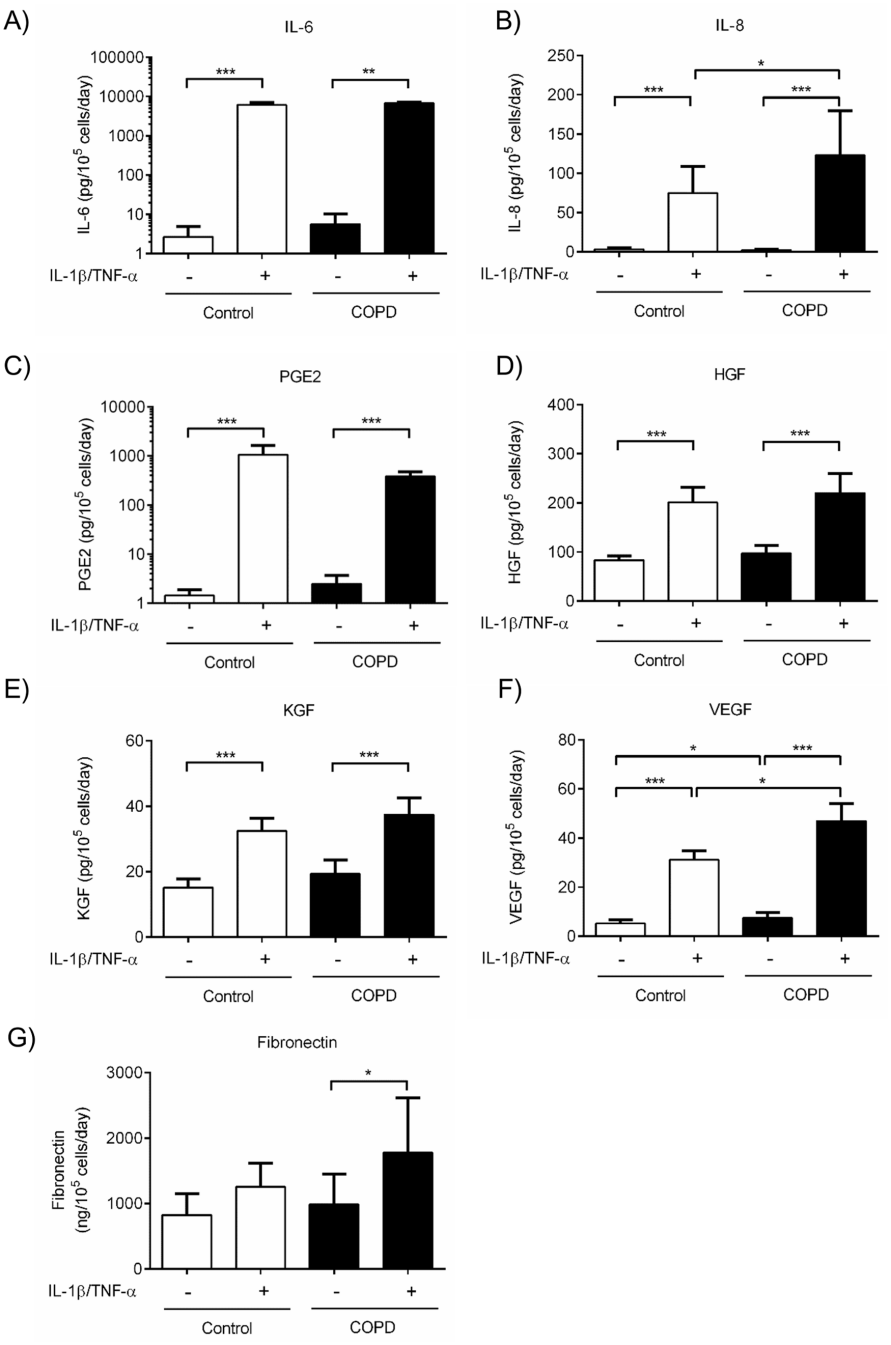
Cytokines, growth factors, lipid mediator and fibronectin release in COPD and control lung fibroblasts. Control (n = 13) and COPD (n = 13) lung fibroblasts were cultured with 10% FCS containing DMEM for 2 days, after which the medium was changed to DMEM in the absence and presence of IL-1ß and TNF-α (1 ng/ml). (A) IL-6, (B) IL-8, (C) PGE_2_, (D) HGF, (E) KGF, (F) VEGF, and (G) fibronectin release in the cultured medium were examined by ELISA or EIA. Vertical axis: (A) IL-6, (B) IL-8, (C) PGE2, (D) HGF, (E) KGF, (F) VEGF release (pg per 10^5^ cells per 1 day) and (G) fibronectin release (ng per 10^5^ cells per 1 day). Horizontal axis: culture condition. White bar: control, Black bar: COPD. *p < 0.05, **p < 0.01, ***p < 0.001.

To clarify a potential role of miR-503 on lung fibroblast function, we first evaluated correlations between miR-503 expression and cytokines, growth factors and ECM production (IL-6, IL-8, PGE2, HGF, KGF, VEGF-A (VEGF) and fibronectin) from primary adult lung fibroblast. Under basal conditions, miR-503 expression and IL-6 (S2A Fig), IL-8 (Fig 4A), PGE2 (Fig 4C), HGF (S2C Fig) and VEGF (Fig 4E), but not KGF (S2E Fig) nor fibronectin (S2G Fig) release were significantly correlated. In the presence of IL-1ß and TNF-α, miR-503 and IL-8 (Fig 4B), HGF (S2D Fig), KGF (S2F Fig) and VEGF (Fig 4F), but not IL-6 (S2B Fig), PGE2 (Fig 4D) nor fibronectin (S2H Fig) release were significantly correlated. Among these mediators, only IL-8 and VEGF but not IL-6, PGE2, HGF, KGF nor fibronectin were produced in larger amount by COPD lung fibroblasts than by control lung fibroblasts either in the absence or presence of IL-1ß and TNF-α (Fig 5A–5G). As expected, cell proliferation of COPD lung fibroblasts, determined by cell number, was significantly reduced compared to that of control lung fibroblasts (S3 Fig).

### MiR-503 inhibits VEGF release from primary control and COPD lung fibroblasts in the presence and absence of exogenous stimuli

**Fig 6:**
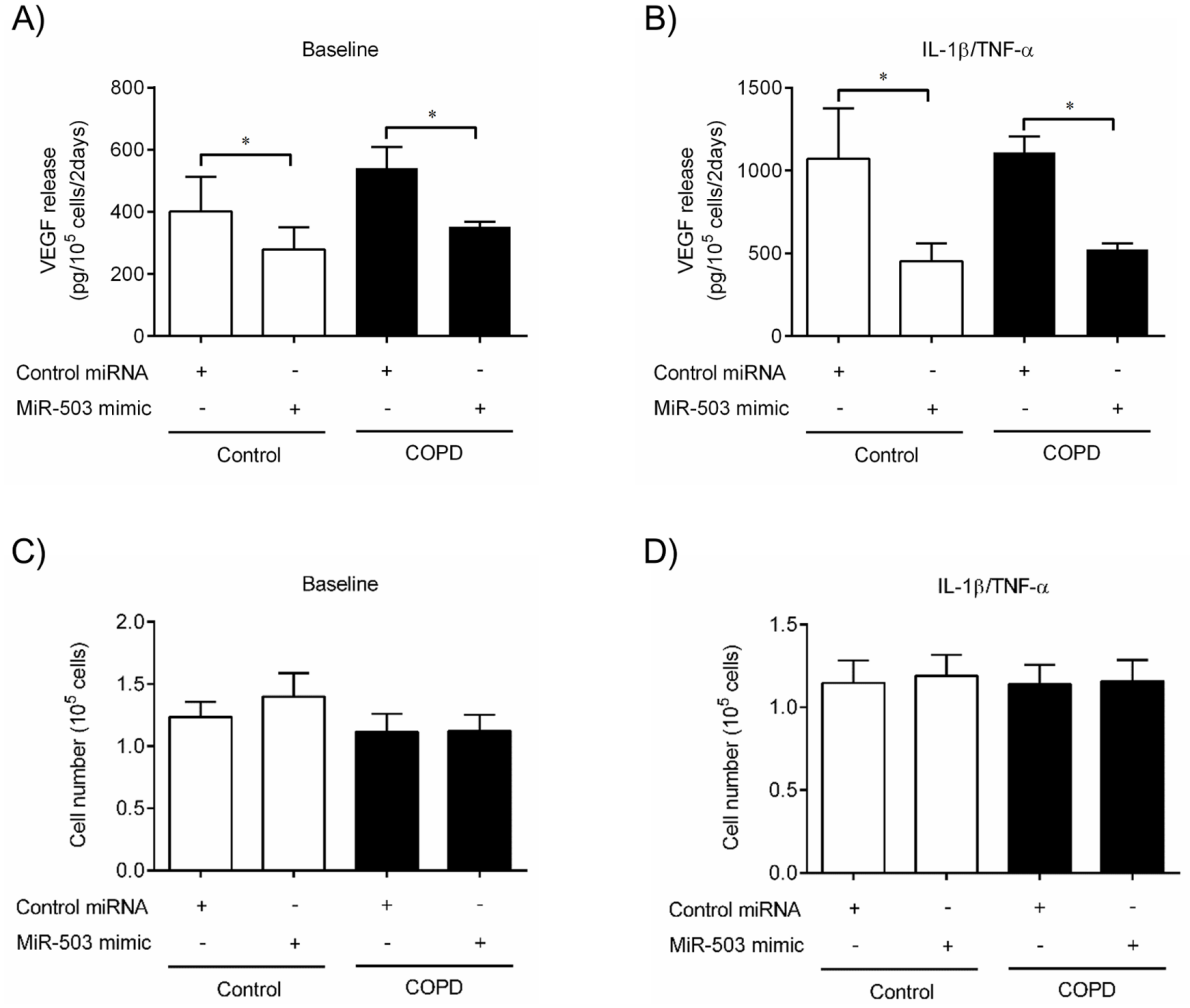
MiR-503 inhibits VEGF production from control and COPD lung fibroblasts. Primary adult control (White bar) and COPD (Black bar) lung fibroblasts (n = 4, respectively) cultured in monolayer were transfected with miR-503 mimic and control transfection reagent, as described in Materials and Methods. 24hr after transfection, the medium was changed to DMEM containing 0.2% FCS, (A, C) without (baseline) or (B, D) with IL-1ß and TNF-α (1 ng/ml). (A, B) After 2 days, the culture medium was harvested and assayed for VEGF by ELISA. Vertical axis: VEGF release (pg per 10^5^ cells per 2 days). (C, D) Cell number after stimulation (10^5^). Horizontal axis: culture condition. *p < 0.05 compared with the values of control miRNA in the same group.

Next, we explored the potential genes that miR-503 might directly target by bioinformatic analysis using online databases (www.targetscan.org; www.mirbase.org). HGF, KGF and VEGF but not IL-6, IL-8, PTGS1 nor PTGS2 (essential genes for PGE_2_ synthesis) gene have a binding site for miR-503. Since miR-503 expression and VEGF were inversely associated, and VEGF release was augmented in COPD lung fibroblasts compared to those in control, we directly tested the hypothesis that decreased miR-503 caused the augmentation of VEGF release in COPD lung fibroblasts. To accomplish this, we first examined whether exogenous miR-503 could alter the production of VEGF in primary lung fibroblasts from individuals with or without COPD. We transfected miR-503 mimic, or negative control miRNA (25 nM, respectively) into control and COPD lung fibroblasts and then measured VEGF release in the presence or absence of IL-1ß and TNF-α (each at 1 ng/ml) 48 hours after stimulation. Compared to negative control miRNA treated cells, miR-503 mimic significantly inhibited VEGF production from both control and COPD lung fibroblasts in basal condition (*p* < 0.05, respectively) (Fig 6A). In the presence of IL-1ß and TNF-α, which stimulated VEGF release, miR-503 repressed VEGF production by more than 2 fold in both control and COPD lung fibroblasts (*p* < 0.05, respectively) (Fig 6B). However, miR-503 transfection did not alter cell number after stimulation with and without IL-1ß and TNF-α in both control and COPD lung fibroblasts (Fig 6C and 6D). Thus, these results suggest that miR-503 inhibits VEGF production from primary control and COPD lung fibroblasts both in the presence and absence of IL-1ß and TNF-α. Moreover, this effect is selective for VEGF production and other altered functions present in COPD fibroblasts are unaffected.

### MiR-503 inhibits VEGF production from human lung fibroblasts by direct binding to the 3’ untranslated region (UTR) of VEGF mRNA

**Fig 7:**
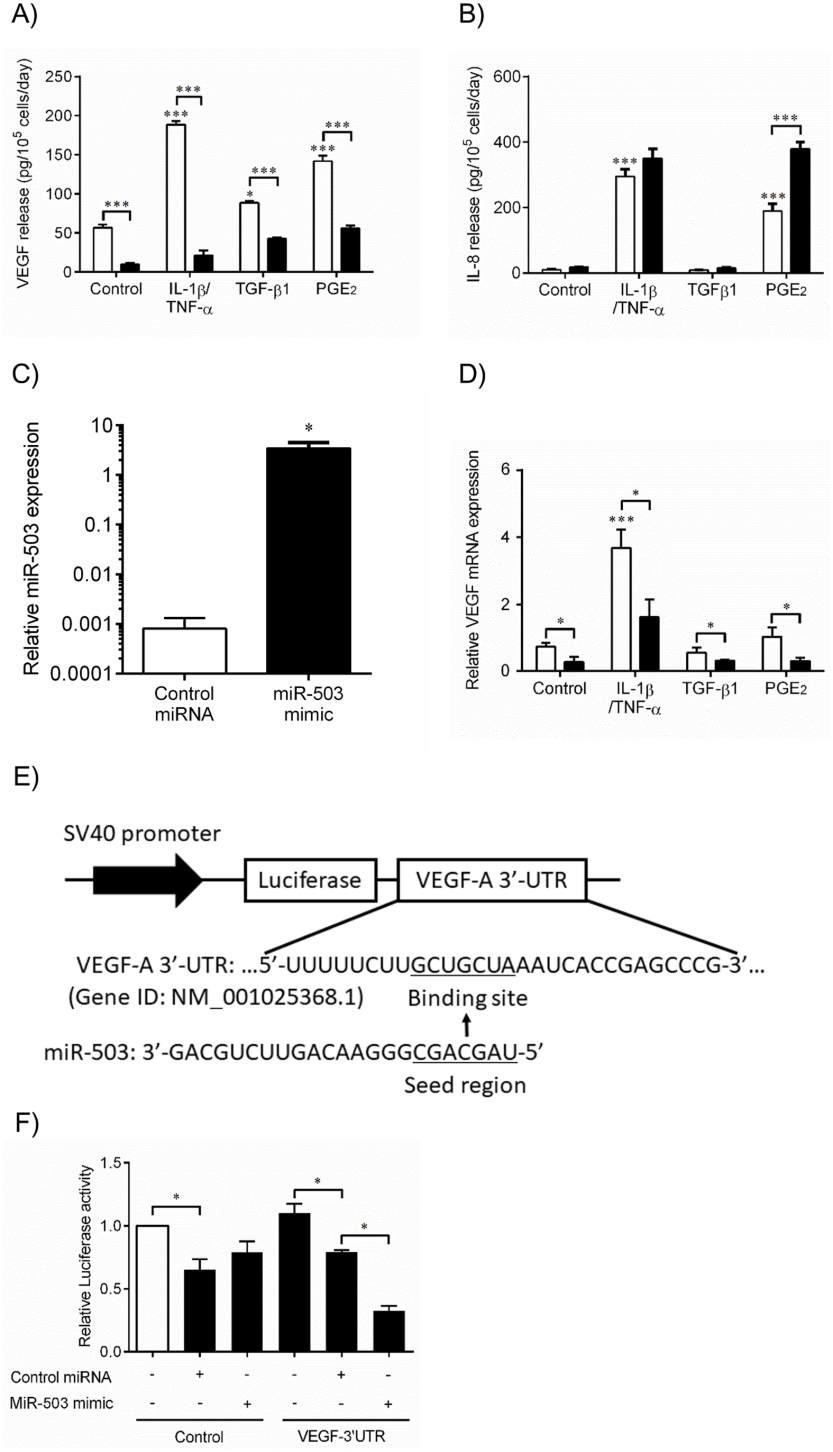
MiR-503 inhibits VEGF protein release and mRNA expression of human lung fibroblasts by direct binding to the 3’ untranslated region (UTR) of VEGF mRNA. (A-D) Primary human fetal lung fibroblasts (HFL-1 cells) cultured in monolayer were transfected with miR-503 mimic (Black bar) or control miRNA (White bar) transfection reagent, as described in Materials and Methods. 24hr after transfection, the medium was changed to DMEM containing 0.2% FCS, with or without IL-1ß and TNF-α (1 ng/ml), TGF-ß1 (1 ng/ml), or PGE_2_ (1 x 10^−7^ M). (A, B) 3 days after transfection, the culture medium was harvested and assayed for (A) VEGF or (B) IL-8 (with or without IL-1ß and TNF-α) by ELISA. (A) Vertical axis: VEGF release (pg per 10^5^ cells per 2 days). (B) Vertical axis: IL-8 release (pg per 10^5^ cells per 2 days). Horizontal axis: culture condition. (C) 1 day after transfection, RNA was isolated and endogenous miR-503 expression was analyzed in the presence of control miRNA or miR-503 mimic by real-time qPCR. Vertical axis: level of miR503 expression, expressed as fold of 18s-rRNA values. (D) 2 days after transfection, RNA was isolated and assayed for VEGF mRNA by real-time qPCR. Vertical axis: level of VEGF mRNA expression, expressed as fold of 18s-rRNA values. Horizontal axis: culture conditions. *p < 0.05, ***p < 0.001. (E) Diagrams showing luciferase reporter constructs containing UTRs of human VEGF-A gene and miR-503 targeting site, and seed and full sequences of miR-503. (F) HFL-1 cells cultured in monolayer were co-transfected with a VEGF 3’ UTR-LUC construct, control vector, miR-503 mimic, and control miRNA. Cell layers were harvested 48hr after transfection, and luciferase activity was analyzed by dual luciferase assay. Vertical axis: Firefly luciferase activity, normalized to Renilla Luciferase activity expressed as a relative value to control (indicated as an open bar). Horizontal axis: culture conditions. *p < 0.05. The data presented are means ± SE from 3 separate experiments.

We sought to examine the molecular mechanisms by which miR-503 inhibits VEGF production by using human fetal lung fibroblasts-1 cells (HFL-1). Since human lung fibroblasts produce increased amounts of VEGF in the presence of several mediators, including TGF-ß1 and PGE_2_, which are highly associated with pathogenesis of COPD [8, 25], we determined if the effect of miR-503 would be observed in the presence of each of these stimuli. Consistent with previous studies [26–28], IL-1ß and TNF-α (*p* < 0.001), TGF-ß1 (*p* < 0.05) and PGE_2　_(*p* < 0.001) significantly augmented VEGF production compared to control media in the presence of negative control miRNA (Fig 7A). Compared to negative control miRNA, the miR-503 mimic significantly inhibited VEGF production under all conditions (control media (*p* < 0.001), IL-1ß/TNF-α (*p* < 0.001), TGF-ß1 (*p* < 0.001) and PGE_2_ (*p* < 0.001)). However, miR-503 mimic did not affect IL-8 release in the absence or presence of IL-1ß and TNF-α. In the presence of PGE2, miR-503 augmented IL-8 release (Fig 7B).

Next, to analyze the effect of miR-503 on VEGF mRNA expression in lung fibroblasts, we transfected HFL-1 cells with negative control miRNA or miR-503 mimic (25 nM) and measured VEGF gene expression in the presence of IL-1ß and TNF-α (1 ng/ml), TGF-ß1 (1 ng/ml), and PGE_2_ (1 x 10^−7^ M) by real time qPCR. First, we confirmed that miR-503 induction was successfully performed by miR-503 mimic transfection (Fig 7C). In HFL-1 cells transfected with negative control miRNA, IL-1ß and TNF-α significantly induced VEGF mRNA expression 24 hours after stimulation, while TGF-ß1 and PGE_2_ did not stimulate VEGF mRNA expression. Compared to negative control miRNA, miR-503 mimic significantly inhibited the VEGF mRNA expression independent of stimulants (Fig 7D). These results indicate that miR-503 inhibits VEGF mRNA expression in the presence of several stimuli that stimulate VEGF production.

Since miR-503 inhibited VEGF regardless of the stimulants, we assessed whether miR-503 directly bound VEGF mRNA. To accomplish this, we used a Luciferase reporter system to evaluate direct binding of miR-503 to the 3’-UTR of the VEGF mRNA. Luciferase vectors containing either UTR region of VEGF-A gene that involves predicted miR-503 binding sequence or control sequence down-stream of the luciferase coding region were used (Fig 7E). Cultured HFL-1 cells were co-transfected with a VEGF 3’ UTR-Luciferase (VEGF-LUC) construct, control vector (Control-LUC), miR-503 mimic, and control miRNA. Cell layers were harvested 48hr after transfection and LUC activity was analyzed. Firefly LUC activity was standardized to Renilla LUC. MiR-503 mimic did not reduce the Control-LUC activity, compared to control miRNA. However, miR-503 significantly reduced VEGF-LUC activity compared to control miRNA (*p* < 0.05) (Fig 7F). Thus, miR-503 directly binds to the VEGF gene and reduces its translation. Taken together, these results indicate that miR-503 regulates both VEGF mRNA and protein levels by directly binding to the VEGF mRNA 3’UTR region in human lung fibroblasts. Through these mechanisms, miR-503 inhibits VEGF release from lung fibroblasts.

### Endogenous expression of miR-503 and effect on VEGF production in HFL-1 cells

**Fig 8:**
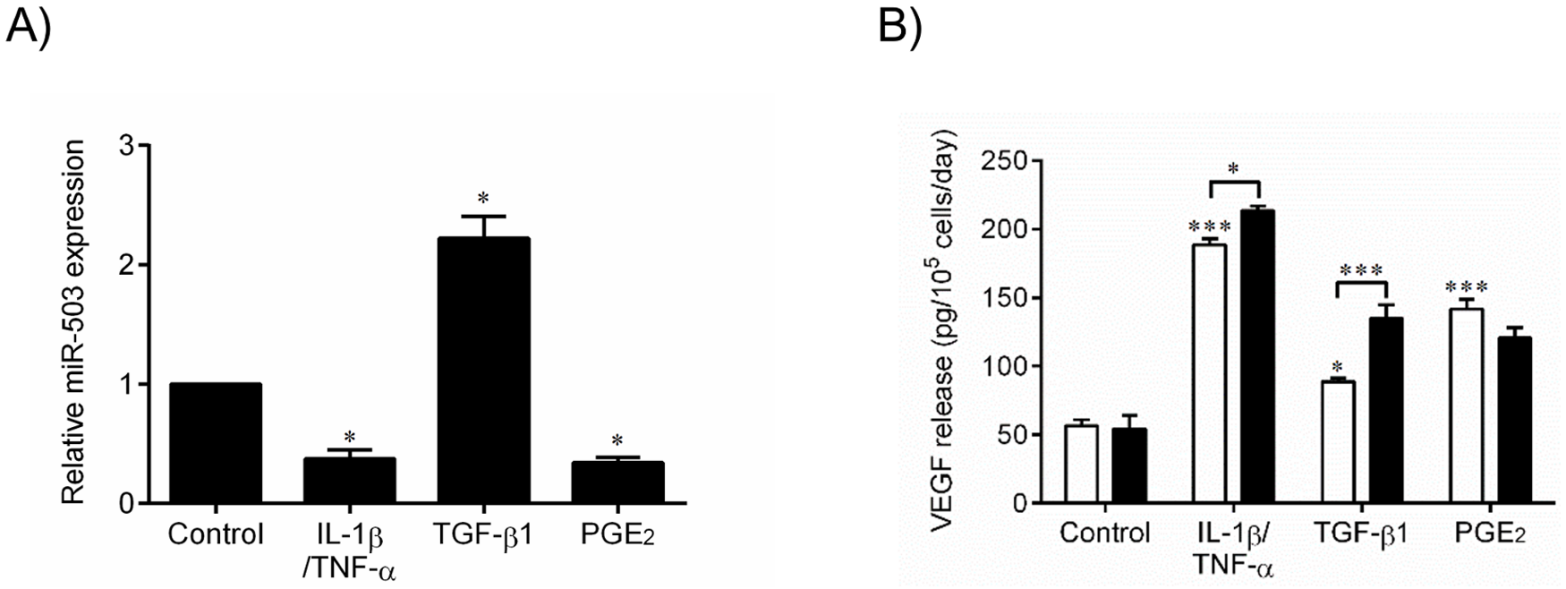
Endogenous miR-503 and VEGF production with various stimulations. HFL-1 cells were cultured in monolayer for 2 days, after which the medium was changed to DMEM containing 0.2% FCS, with or without IL-1ß and TNF-α (1 ng/ml), TGF-ß1 (1 ng/ml) or PGE_2_ (1 x 10^−7^ M). (A) 1 day after stimulation, RNA was isolated and miR-503 expression was analyzed by real-time qPCR. MiR-503 expression was calculated as fold of 18s-rRNA expression. Vertical axis: level of miR-503 expression, expressed as fold of control medium alone. Horizontal axis: culture conditions. *p < 0.05 compared with the values of control medium alone. (B) HFL-1 cells cultured in monolayer were transfected with miR-503 inhibitor (Black bar) or control miRNA (White bar) transfection reagent, as described in Materials and Methods. 24hr after transfection, the medium was changed to DMEM containing 0.2% FCS, with or without TGF-ß1 (1 ng/ml), IL-1ß and TNF-α (1 ng/ml) or PGE_2_ (1 x 10^−7^ M). 3 days after transfection, the culture medium was harvested and assayed for VEGF by ELISA. Vertical axis: VEGF release (pg per 10^5^ cells per 2 days). *p < 0.05, ***p < 0.001 compared with the values of control medium alone. The data represent the means ± SE of 3 separate experiments.

To further characterize the role of miR-503 expression in VEGF release modulated by different stimuli, we stimulated HFL-1 cells with control media, IL-1ß and TNF-α (each at 1 ng/ml), TGF-ß1 (1 ng/ml), or PGE_2_ (1 x 10^−7^ M) for 24 hours and assessed miR-503 expression by real time qPCR. Consistent with the results in primary adult lung fibroblasts, IL-1ß and TNF-α significantly suppressed miR-503 expression. However, TGF-ß1 significantly stimulated miR-503, whereas PGE_2_ inhibited its expression. These results suggest that the endogenous expression of miR-503 is differentially regulated by different exogenous stimuli (Fig 8A). Next, we examined the effect of endogenous miR-503 inhibition on VEGF release by human lung fibroblasts with and without various stimulants. MiR-503 inhibitor increased VEGF production compared to negative control miRNA in the presence of IL-1ß and TNF-α (*p* < 0.05), and TGF-ß1 (*p* < 0.001). However, miR-503 inhibitor did not affect VEGF release in basal conditions or in the presence of PGE_2_ (Fig 8B). Thus, the role of endogenous miR-503 on VEGF release from lung fibroblasts may vary in the presence of different extracellular mediators.

## Discussion

In the current study, we confirmed the observation that miR-503 expression was significantly decreased in COPD lung fibroblasts using real-time qPCR in a larger group of COPD and control subjects. Importantly, miR-503 expression significantly correlated with lung function, pulmonary arterial pressure. We then demonstrated that miR-503 expression correlated with IL-6, -8, PGE2, HGF, KGF, and VEGF release from lung fibroblasts and that IL-8 and VEGF release from COPD lung fibroblasts were increased compared to those from control in the absence or presence of IL-1ß and TNF-α. We examined the regulation of VEGF release in response to several mediators believed to play roles in COPD and demonstrated that miR-503 regulates VEGF production in the presence of all tested. Furthermore, exogenous miR-503 repressed both VEGF mRNA and protein expression and directly binds to the 3’-UTR region of the VEGF mRNA. Endogenous miR-503 was differently regulated by exogenous stimulants, IL-1ß and TNF-α, TGF-ß1 and PGE2 and demonstrated selectivity of miR-503 regulation of VEGF. Thus, the results of the present study suggest that altered miR-503 expression in lung fibroblasts may play a role in vascular homeostasis in COPD.

MiRNAs have drawn attention in several lung diseases, including COPD [14, 15, 29]. However, understanding of the precise role of specific miRNAs in defined cell types, including lung fibroblasts, is limited [10]. Formerly, we identified differentially expressed miRNAs in COPD lung fibroblasts *in vitro* [16]. Among the differentially expressed miRNA species, miR-503 expression was significantly reduced in cultured COPD lung fibroblasts under both basal and IL-1ß and TNF-α as assessed by microarray analysis. MiR-503 is involved in several biologic and pathologic processes, including cell cycle, angiogenesis and cancer growth [30, 31]. Consistent with this, the seed region of miR-503 is evolutionarily conserved among different species [30]. In addition, miR-503 is abundantly expressed in the lung, and is associated with pulmonary hypertension [17]. However, the role of miR-503 in COPD pathogenesis, especially in lung fibroblasts, has been unclear. Previously, we demonstrated that one type of miRNA, miR-146a, is decreased in COPD lung fibroblasts and this causes increased production of an inflammatory mediator, prostaglandin (PG) E_2_, by reduced targeting of COX-2 mRNA after stimulation with IL-1β and TNF-α. Since PGE_2_ is increased in the lower airway of patients with COPD [32–34] and inhibits the repair function of lung fibroblasts [35, 36], these results support a role for altered miRNA expression in lung fibroblasts contributing to the pathogenesis of COPD. Thus, the current study extends these results suggesting that altered fibroblast miR-503 expression may also contribute to the pathogenesis of COPD.

As functions of lung fibroblasts are altered in COPD in multiple ways, including extracellular mediator production, gel contraction, chemotaxis and cell proliferation [8–11], we examined the role of miR-503 in modulating the production of IL-6, -8, PGE_2_, HGF, KGF, VEGF and fibronectin, all of which are associated with COPD pathogenesis [8, 11, 18–21]. Among these mediators, IL-6, IL-8, PGE_2_, HGF, KGF and VEGF were associated with miR-503 expression, either in the absence or presence of IL-1ß and TNF-α, suggesting that miR-503 might orchestrate several lung fibroblasts secretory functions. By using established bioinformatic databases, HGF, KGF and VEGF but not IL-8 gene were identified as having potential binding sites for miR-503. Because VEGF, a key modulator of vascular cells, was produced in greater amounts from COPD lung fibroblasts compared to control subject fibroblasts both in the presence and absence of IL-1ß and TNF-α, we focused the remainder of our studies on defining a role for miR-503 in regulating VEGF. In this context, exogenous miR-503 significantly suppressed VEGF production in primary lung fibroblasts. Although IL-8 release was augmented in COPD lung fibroblasts as compared to that of control, exogenous miR-503 did not inhibit IL-8 release in human fetal lung fibroblasts, supporting the concept that IL-8 is not be a direct target of miR-503 in lung fibroblasts. These results suggest that, in COPD, decreased miR-503 may specifically contribute to the augmentation of VEGF release by human lung fibroblasts in basal and inflammatory conditions. Consistent with the prior study [9], COPD lung fibroblasts showed reduced proliferation rate in vitro. Exogenous miR-503 did not affect cell proliferation in either control or COPD lung fibroblasts. A range of functions including gel contraction and chemotaxis are altered in COPD lung fibroblast [8]. Whether these are affected by miR-503 was not examined in the current study. What the current study demonstrates, however, is that altered expression of miR-503 accounts for some, but not all the altered functions of COPD fibroblasts. In that context, we have previously shown that altered production of miR-146a also accounts for alterations in some functions of COPD fibroblasts [10]. This suggests that altered function of COPD fibroblasts depends on multiple alterations in a complex signaling network.

VEGF is a potent growth factor regulating vasculature and its expression is often excessive in chronic inflammation [22]. VEGF is inducible by several inflammatory cytokines associated with COPD pathogenesis, including IL-1ß and TNF-α [22]. Furthermore, the fibroblast is a major producer of VEGF in inflamed tissue [37]. Previously, we have reported that lung fibroblast produce VEGF and among their other potential roles in the pathogenesis of COPD [23, 26, 38]. In this context, vascular homeostasis is disturbed in COPD, and several human and animal studies have assessed a potential role for VEGF in COPD pathogenesis [22]. Kasahara and colleagues reported that extractable VEGF and its receptor were reduced in the lungs of severe COPD patients compared to control [20]. Further, antagonism of VEGF signaling can lead to the development of emphysema in animal models [39]. In contrast, Kranenburg et al. reported increased VEGF in the lungs of patients with moderate COPD, with increased expression in the bronchiolar and alveolar epithelium as well as the smooth muscle of small bronchioles and arterioles. Moreover, the increased VEGF expression in lung tissue was related to decreased lung function [40]. This is analogous to the results of the current study, which found increased fibroblast VEGF production in COPD. Interestingly, Santos et al. reported increased extractable VEGF and *in situ* immunohistochemical staining in smokers and patients with moderate COPD compared to controls, but reduced amounts in patients with severe COPD [41]. The patients in the current study generally had severe or very severe COPD. These results raise the possibility that there may be different levels of VEGF expression at different stages of COPD. Similarly, VEGF produced by different cells may play different pathogenic roles at different sites within the lung. Resolving these questions will require studies of a different design than those conducted to date. Nevertheless, the available studies all support a role for VEGF in the pathogenesis of COPD. We could not find statistical association between VEGF release and clinical severity of COPD in the current data set (data not shown). Thus, the exact role of miR-503-VEGF-axis in fibroblasts *in vivo* remains to be elucidated, and it is likely that the overall effect will depend on complex signaling networks. In this context, miR-503 also targets the growth factor FGF2 and its receptor FGR1. Decreased expression of miR-503 has been suggested to have a pathogenic role in pulmonary artery hypertension by increasing expression of these proteins [17]. Thus, to what extent interactions among various targets of miR-503 play roles in COPD pathogenesis remains to be resolved.

The current study elucidated the molecular mechanisms of VEGF regulation by miR-503 by using HFL-1 cells. HFL-1 is a primary fetal lung fibroblast line which has been widely used for *in vitro* research because of its reliable growth and stability. Use of this strain is also helpful as it allows comparison of results across different laboratories. IL-1ß, TNF-α and TGF- ß contribute to lung inflammation and fibrosis in COPD [25]. PGE_2_ play an important role in disturbed repair function in COPD [8]. As, these mediators, apart from TNF-α alone, augment VEGF release from human lung fibroblasts [24, 26], we sought to examine if the effect of miR-503 would be observed in response to multiple stimuli: IL-1ß and TNF-α, TGF- ß and PGE_2_. We demonstrated VEGF mRNA and protein expression was suppressed by miR-503 and that this occurred in the presence IL-1ß and TNF-α, TGF- ß and PGE_2_. However, the miR-503 did not inhibit IL-8 release but augmented in the presence of PGE2 in protein level. These suggest that miR-503 directly targets VEGF mRNA and is not acting indirectly by modulating other signaling pathways. Consistent with this observation, use of a lucifrerase reporter system confirmed that miR-503 directly binds to the 3’-UTR region of the VEGF mRNA in human lung fibroblasts. Through these mechanisms, miR-503 down-regulate VEGF in response to mediators involved in COPD pathogenesis.

In accordance with our study, Zhou et al. demonstrated that miR-503 directly suppresses VEGF and inhibits tumorgenesis in human hepatocellular carcinoma [30]. In addition, endothelial miR-15a, which has the same seed sequence as miR-503, negatively regulates angiogenesis by suppression of FGF2 and VEGF [42]. Both miR-503-binding sites on FGF2 and VEGF-A are evolutionarily conserved among different mammalian species [30]. Thus, miR-503 could physiologically regulate VEGF and the current study extends these observations to include lung mesenchymal cells and demonstrates a relationship between miR-503 and VEGF across a range of expression levels in cells obtained from patients with COPD and controls.

Finally, having shown that miR-503 can directly modulate VEGF by binding VEGF mRNA, we sought to determine a role for miR-503 in mediating the effect of various stimuli on VEGF production. In this context, TGF-ß1 significantly augmented miR-503. In contrast, IL-1ß and TNF-α, and PGE_2_ reduced miR-503 expression. Interestingly, the effect of endogenous miR-503 in modulating VEGF was most prominent in the presence of TGF-ß1 as compared to baseline, IL-1ß and TNF-α or PGE_2_. Thus, reduction of endogenous miR-503 appears to mediate stimulation of VEGF production by IL-1ß and TNF-α, and PGE_2_, but increases in miR-503 appear to play a feedback role limiting stimulation by TGF-ß1.

Primarily because of limitations of transcriptional methodology, the mechanisms underlying miRNA regulation remains poorly understood [43]. However, many miRNAs are thought to use their own transcription initiation regions, e.g. a stretch of DNA between clusters of genes (intergenic) or embedded within introns of known coding genes (intronic) [43]. Although the mechanisms that regulate miR-503 transcription and maturation have not yet been defined [31], previous studies demonstrated that several exogenous and endogenous factors, including phorbol myristate acetate, apelin and HIF-1α affect miR-503 expression [44]. Thus, the current study extends the mediators that modulate miR-503.

There are several limitations to the current study. First, the smoking status of control and COPD donors from whom lung fibroblasts were cultured differed. Since smoking is reported to have effects on miRNA expression [45, 46], it is possible that the reduced miR-503 expression in COPD lung fibroblasts was due to differences in smoking status. Second, as the control group includes fibroblasts from unused lungs from organ donor patients, some of the clinical data, such as lung function tests, were lacking. This limits the power of the analysis of the relationship between clinical data and miR-503. Third, miR-503 may regulate various target genes other than VEGF, which were not fully assessed in the current study. Thus, the full pathophysiological role of miR-503 remains to be defined. Finally, the mechanisms by which miR-503 is down-regulated in the COPD lung fibroblasts remain to be elucidated.

In conclusion, the current study demonstrates differential expression of miR-503 in the COPD lung and that miR-503 may play a pathogenic role in vascular homeostasis in COPD. Since therapies using miRNA analogues are being developed, the miR-503/VEGF axis may be a potential therapeutic candidate for the treatment of COPD

## Supporting information

S1 Fig Correlations between clinical data and miR-503 expression in control and COPD lung fibroblasts cultured with or without IL-1ß and TNF-α. Control (n = 19) and COPD (n = 18) lung fibroblasts were cultured with 10% FCS containing DMEM for 2 days, after which the medium was changed to DMEM in the absence (baseline) or presence of IL-1ß/TNF-α (1 ng/ml). After 1 day, total RNA was extracted from the cultured cells. Correlation between miR-503 expression in lung fibroblasts and mean pulmonary arterial pressure (mPAP (mmHg)) (Control (n = 0) and COPD (n = 12)) ((A) baseline, (B) IL-1ß/TNF-α), Age (Control (n = 16) and COPD (n = 17)) ((C) baseline, (D) IL-1ß/TNF-α), smoking (Pack-Year) (Control (n = 4) and COPD (n = 11)) ((E) baseline, (F) IL-1ß/TNF-α), or 6-minutes walking distance (6MWD (m)) (Control (n = 0) and COPD (n = 12)) ((G) baseline, (H) IL-1ß/TNF-α) were shown. White square: control, Black triangle: COPD. Horizontal axis: level of miR-503 expression, expressed as fold of 18s-rRNA values. The correlation was calculated by Spearman’s correlation test.
(TIF)

S2 Fig IL-6, HGF, KGF and fibronectin release and their correlations with miR-503 expression in COPD and control lung fibroblasts. Control (n = 13) and COPD (n = 13) lung fibroblasts were cultured with 10% FCS containing DMEM for 2 days, after which the medium was changed to DMEM in the absence and presence of IL-1ß and TNF-α (1 ng/ml). After 1 day, the cell layer was harvested and miR-503 expression was examined by real-time qPCR. IL-6, HGF, KGF, and fibronectin release in the cultured medium were examined by ELISA or EIA. The correlation between miR-503 expression and IL-6 ((A) baseline, (B) IL-1ß/TNF-α), HGF ((C) baseline, (D) IL-1ß/TNF-α), KGF ((E) baseline, (F) IL-1ß/TNF-α), and fibronectin ((G) baseline, (H) IL-1ß/TNF-α) are shown. White square: control, Black triangle: COPD. Vertical axis: IL-6, HGF, KGF release (pg per 10^5^ cells per 1 day) and fibronectin release (ng per 10^5^ cells per 1 day), respectively. Horizontal axis: level of miR-503 expression, expressed as fold of 18s-rRNA values. The correlation was calculated by Spearman’s correlation test.
(TIF)

S3 Fig Cell number in COPD and control lung fibroblasts. Control (n = 13) and COPD (n = 13) lung fibroblasts were cultured with 10% FCS containing DMEM for 2 days, after which the medium was changed to DMEM in the absence and presence of IL-1ß and TNF-α (1 ng/ml). Cell number was examined after stimulation (10^6^). Horizontal axis: culture condition. White bar: control, Black bar: COPD. *p < 0.05.
(TIF)
