# Supplementary figures and images for: Reduced microRNA-503 expression augments lung fibroblast VEGF production in chronic obstructive pulmonary disease
Source: PLoS One. 2017 Sep 7;12(9):e0184039. doi: 10.1371/journal.pone.0184039 (PMC5589164; doi:10.1371/journal.pone.0184039)

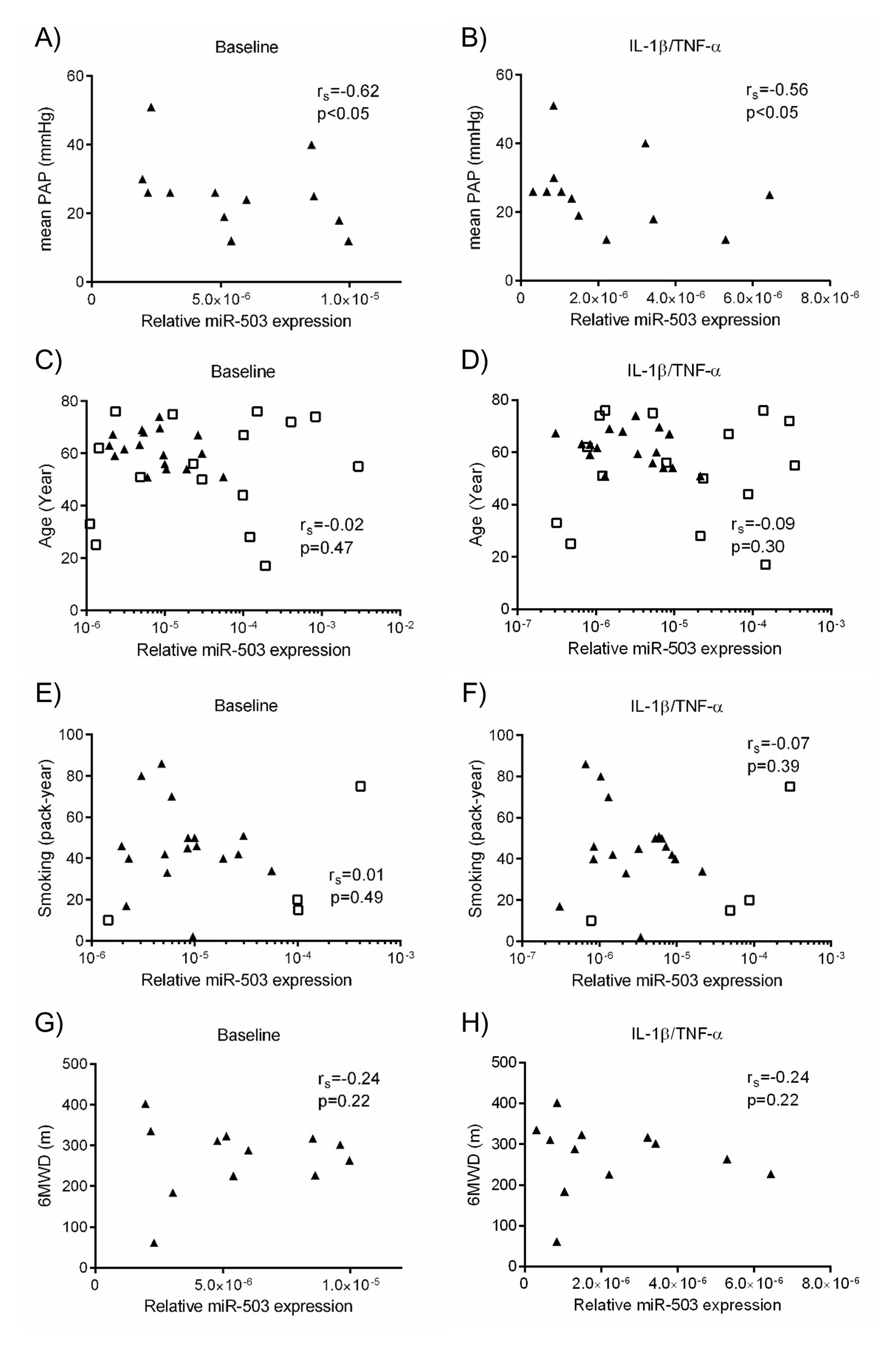

Supplement: S1 Fig — Control (n = 19) and COPD (n = 18) lung fibroblasts were cultured with 10% FCS containing DMEM for 2 days, after which the medium was changed to DMEM in the absence (baseline) or presence of IL-1ß/TNF-α (1 ng/ml). After 1 day, total RNA was extracted from the cultured cells. Correlation between miR-503 expression in lung fibroblasts and mean pulmonary arterial pressure (mPAP (mmHg)) (Control (n = 0) and COPD (n = 12)) ((A) baseline, (B) IL-1ß/TNF-α), Age (Control (n = 16) and COPD (n = 17)) ((C) baseline, (D) IL-1ß/TNF-α), smoking (Pack-Year) (Control (n = 4) and COPD (n = 11)) ((E) baseline, (F) IL-1ß/TNF-α), or 6-minutes walking distance (6MWD (m)) (Control (n = 0) and COPD (n = 12)) ((G) baseline, (H) IL-1ß/TNF-α) were shown. White square: control, Black triangle: COPD. Horizontal axis: level of miR-503 expression, expressed as fold of 18s-rRNA values. The correlation was calculated by Spearman’s correlation test. (TIF) [file pone.0184039.s001.tif]

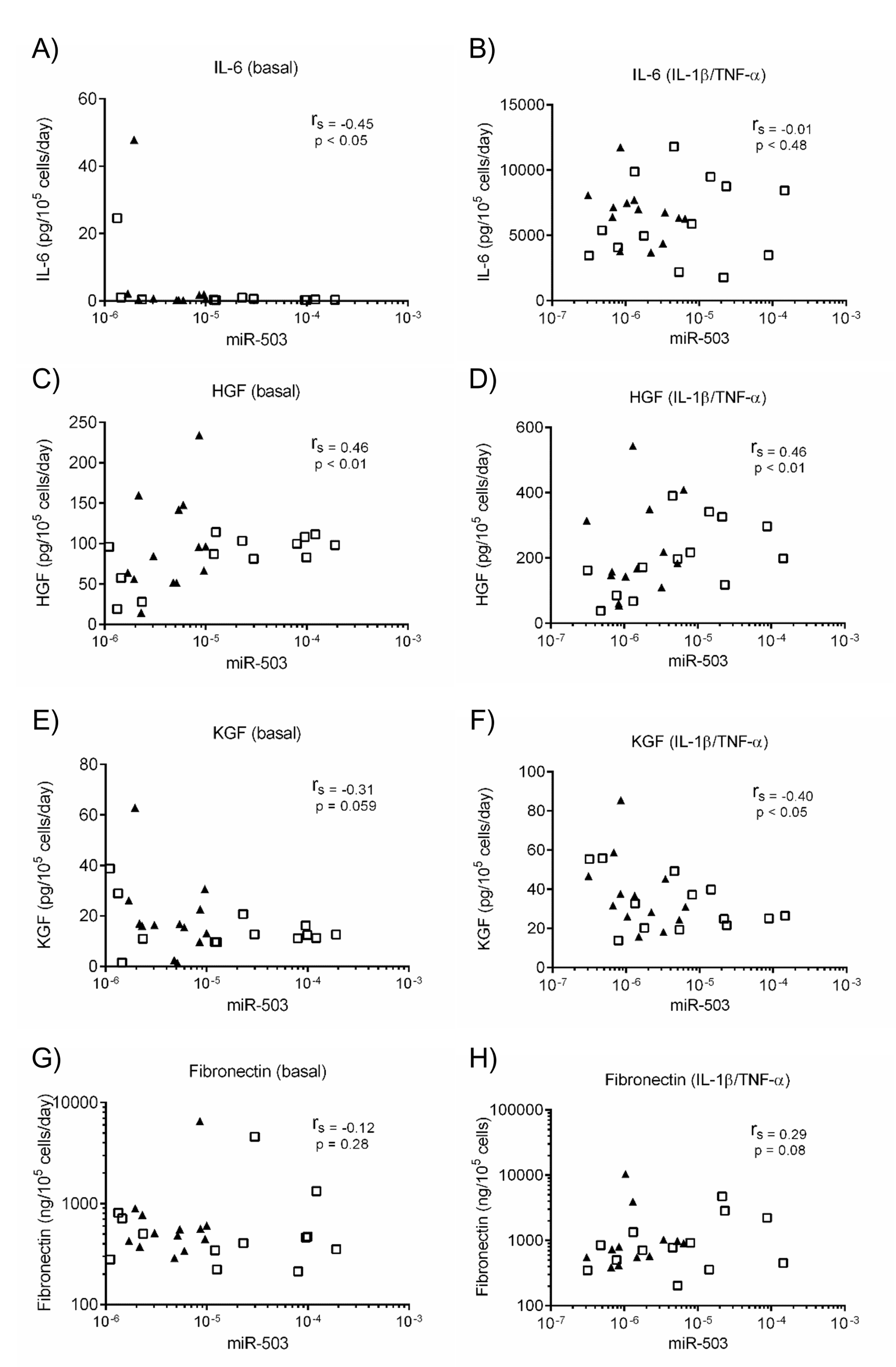

Supplement: S2 Fig — Control (n = 13) and COPD (n = 13) lung fibroblasts were cultured with 10% FCS containing DMEM for 2 days, after which the medium was changed to DMEM in the absence and presence of IL-1ß and TNF-α (1 ng/ml). After 1 day, the cell layer was harvested and miR-503 expression was examined by real-time qPCR. IL-6, HGF, KGF, and fibronectin release in the cultured medium were examined by ELISA or EIA. The correlation between miR-503 expression and IL-6 ((A) baseline, (B) IL-1ß/TNF-α), HGF ((C) baseline, (D) IL-1ß/TNF-α), KGF ((E) baseline, (F) IL-1ß/TNF-α), and fibronectin ((G) baseline, (H) IL-1ß/TNF-α) are shown. White square: control, Black triangle: COPD. Vertical axis: IL-6, HGF, KGF release (pg per 105 cells per 1 day) and fibronectin release (ng per 105 cells per 1 day), respectively. Horizontal axis: level of miR-503 expression, expressed as fold of 18s-rRNA values. The correlation was calculated by Spearman’s correlation test. (TIF) [file pone.0184039.s002.tif]

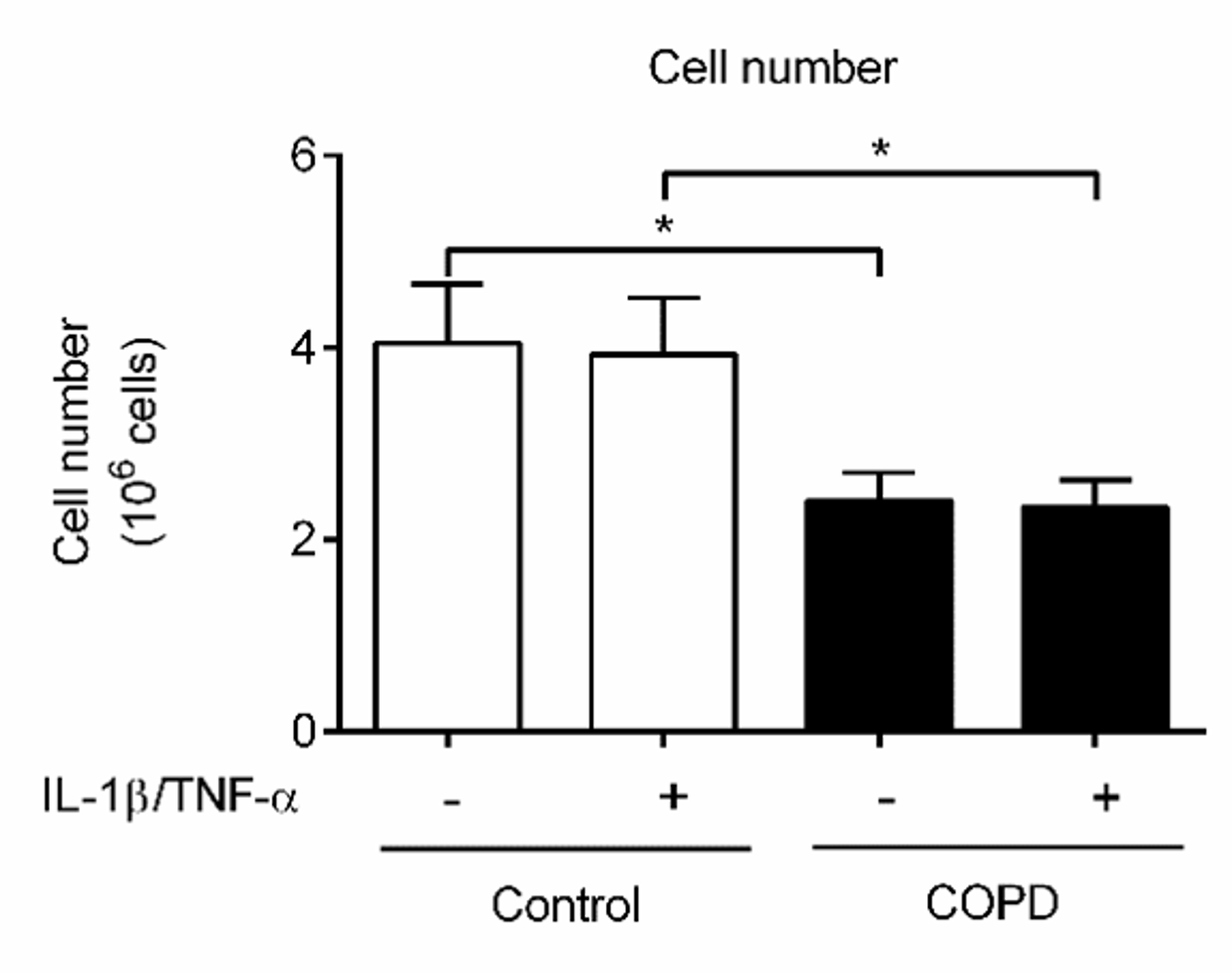

Supplement: S3 Fig — Control (n = 13) and COPD (n = 13) lung fibroblasts were cultured with 10% FCS containing DMEM for 2 days, after which the medium was changed to DMEM in the absence and presence of IL-1ß and TNF-α (1 ng/ml). Cell number was examined after stimulation (106). Horizontal axis: culture condition. White bar: control, Black bar: COPD. *p < 0.05. (TIF) [file pone.0184039.s003.tif]
